# Endometrial Carcinoma Unveiling Breast Carcinoma in a Postmenopausal Female: A Case of Synchronous Malignancy

**DOI:** 10.31486/toj.25.0116

**Published:** 2026

**Authors:** Kalyan Chakradhar Polavarapu, Mada Pooja, Renuka Sri Sai Peddireddi, Suresh K. Alahari

**Affiliations:** ^1^ Department of Surgical Oncology, American Oncology Institute, Guntur, Andhra Pradesh, India; ^2^ Department of Radiation Oncology, NRI Medical College, Guntur, Andhra Pradesh, India; ^3^ Department of Obstetrics and Gynecology, Indira Gandhi Medical College and Research Institute, Puducherry, India; ^4^ Department of Pharmacology, Biochemistry and Experimental Therapeutics LSU-LCMC Cancer Center, Louisiana Cancer Research Center, Louisiana State University Health Sciences Center, New Orleans, LA

**Keywords:** *Breast neoplasms*, *endometrial neoplasms*, *neoplasms–multiple primary*, *positron emission tomography computed tomography*

## Abstract

**Background:**

The ability to detect multiple primary malignancies in a single patient has improved because of modern cancer screening and imaging technologies. While both endometrial and breast carcinomas are common in postmenopausal women, concomitant presentation is uncommon.

**Case Report:**

A 61-year-old postmenopausal female initially presented with abdominal pain and postmenopausal bleeding. Positron emission tomography/computed tomography revealed fluorodeoxyglucose-avid lesions in the left breast and axilla, although mammography showed Breast Imaging Reporting and Data System category 1. Clinical breast and axillary examinations were unremarkable. Immunohistochemistry demonstrated GATA binding protein 3 and human epidermal growth factor receptor 2 positivity in the axillary lymph nodes, suggestive of breast carcinoma, while positivity for paired box gene 8, estrogen receptors, and progesterone receptors supported an endometrial tumor. Genetic testing panel results were negative. After undergoing neoadjuvant chemotherapy, the patient had marked regression in the endometrial lesion and a pathologic complete response in the breast lesion.

**Conclusion:**

Because of the infrequency and clinical complexity associated with synchronous malignancies, this case highlights the importance of a multidisciplinary approach, advanced imaging, and molecular profiling for accurate diagnosis and personalized treatment planning.

## INTRODUCTION

Breast and endometrial cancers are among the most common malignancies affecting postmenopausal females. Epidemiologic studies report that the frequency of multiple primary tumors ranges from 2% to 17%.^1^ Contributing factors include genetic, hormonal, and environmental influences, as well as prior exposure to chemotherapy or radiotherapy that may predispose to a second primary malignancy.^1^

Irimie et al defined the term synchronous cancer as a second primary malignant tumor that occurs simultaneously or within 6 months of the diagnosis of a first primary neoplasm.^2^ The tumors occur in different organs and have different histopathologic features.^2^ Managing synchronous cancers is clinically challenging and requires therapeutic strategies that address both diseases while minimizing toxicity and preserving prognosis.

We present the case of a postmenopausal female with synchronous endometrial endometrioid carcinoma and invasive breast carcinoma.

## CASE REPORT

A 61-year-old postmenopausal female presented in January 2024 with complaints of vaginal bleeding and lower abdominal pain. She had a medical history of diabetes mellitus and hypertension, both well controlled on medication. Her family history was unremarkable. She had 2 children and no history of oral contraceptive use. On examination, the patient was alert and oriented, with a body mass index of 31.1 kg/m^2^. Abdominal examination was normal. Speculum examination revealed a healthy cervix, and bimanual examination showed an anteverted uterus. Both breasts and axillae were clinically unremarkable.

**Figure 1.**
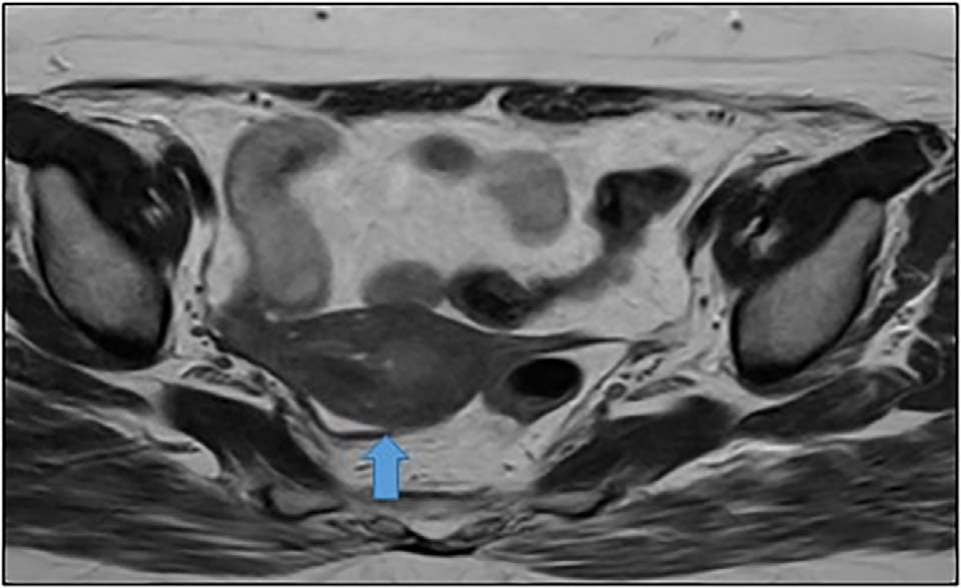
T2-weighted axial magnetic resonance imaging of the pelvis shows a hyperintense polypoidal lesion in the upper endometrium (arrow).

Pelvic magnetic resonance imaging (MRI) revealed a 19 × 15 × 11-mm polypoidal lesion in the upper endometrium that was infiltrating the myometrium, without lymphadenopathy (Figure 1). Histopathology of the endometrial curettings confirmed a well-differentiated endometrioid carcinoma (International Federation of Gynaecology and Obstetrics [FIGO] grade 1).

**Figure 2.**
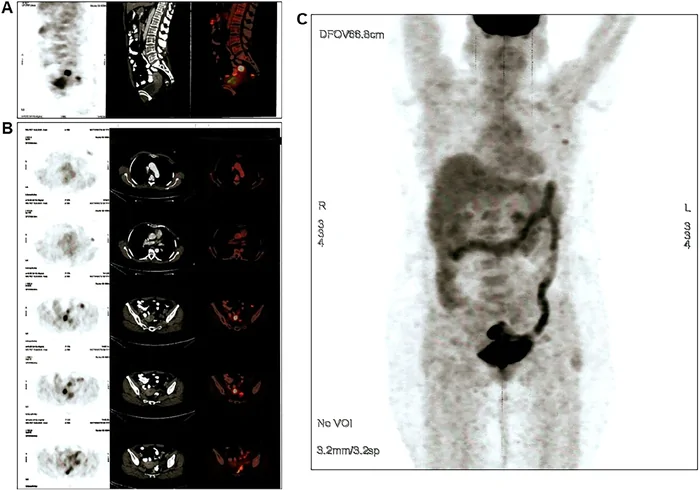
Fluorodeoxyglucose positron emission tomography/computed tomography (A) sagittal view of the pelvis shows increased metabolic activity in the endometrium and myometrium; (B) axial views of the thorax show mild metabolically active small nodules in the upper inner quadrant of the left breast and left axillary nodes; and (C) coronal view of the whole body shows metabolically active lesions in the left breast and left axilla.

Positron emission tomography/computed tomography (PET/CT) revealed a 23 × 16 × 25-mm hypodense endometrial lesion (standard uptake value [SUV] 19.1) and mildly fluorodeoxyglucose (FDG)-avid nodules in the left breast (largest 7 mm; SUV 1.8) with a few enlarged left axillary lymph nodes (largest 12 × 8 mm; SUV 2.1) (Figure 2). However, mammography showed normal bilateral breasts (Breast Imaging Reporting and Data System category 1, American College of Radiology type A).

**Figure 3.**
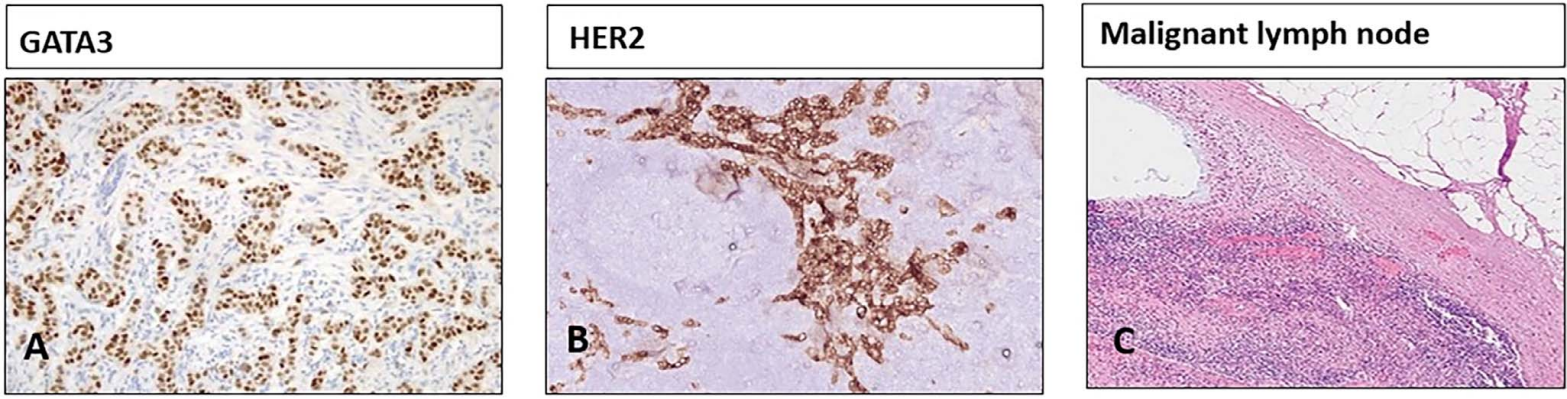
Immunohistochemistry of left axillary lymph nodes shows (A) moderate positivity for GATA binding protein 3 (GATA3) and (B) positivity for human epidermal growth factor receptor 2 (HER2). (C) Hematoxylin and eosin stain shows breast carcinoma.

**Figure 4.**
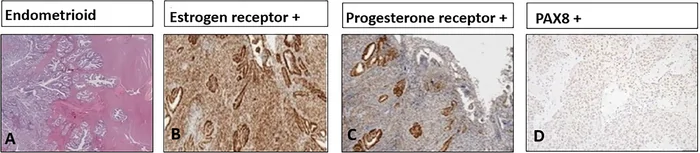
Immunohistochemistry of the endometrial carcinoma shows (A) endometrioid carcinoma with myometrial invasion, (B, C) tumor cells with nuclear staining positive for estrogen receptors and progesterone receptors, and (D) tumor cells weakly positive for paired box gene 8 (PAX8).

Fine needle aspiration cytology of the axillary lymph nodes showed metastatic adenocarcinoma, and biopsy confirmed metastatic adenocarcinoma in 2 of 8 lymph nodes without extranodal spread. Immunohistochemistry confirmed breast carcinoma (GATA binding protein 3 positive and human epidermal growth factor receptor 2 [HER2] positive), and the endometrial lesion expressed paired box gene 8, estrogen receptors, and progesterone receptors consistent with endometrioid carcinoma (Figures 3 and 4, Table 1). Genetic testing panel results were negative.

**Table 1.** Immunohistochemistry Report

Marker	Endometrial Tumor	Left Axillary Nodal Deposit
Estrogen receptors	3 + 5 = 8/8 (Positive)	0 (Negative)
Progesterone receptors	3 + 5 = 8/8 (Positive)	0 (Negative)
Human epidermal growth factor receptor 2	0 (Negative)	3+ (Positive)
Ki-67	4%	5%
Paired box gene 8	Weak positive	Negative
GATA binding protein 3	Negative	Moderate positive
p53	Negative	Positive

Following a multidisciplinary tumor board discussion, treatment was initiated in February 2024. The patient received 3 cycles of neoadjuvant TCH chemotherapy—docetaxel 75 mg/m^2^, carboplatin area under the curve 6, and trastuzumab with a loading dose of 8 mg/kg followed by a maintenance dose of 6 mg/kg administered at 3-week intervals. After 3 cycles of chemotherapy, MRI of the pelvis showed partial regression of the endometrial lesion, and sonomammogram showed no mass lesion in either breast and no axillary lymphadenopathy.

In May 2024, the patient underwent radical hysterectomy with bilateral salpingo-oophorectomy and pelvic lymph node dissection. Histology confirmed endometrioid carcinoma (FIGO grade 1) with a maximum tumor diameter of 8 mm; <50% myometrial invasion; no lymphovascular space invasion; and uninvolved cervix, margins, and lymph nodes (FIGO stage IA2).

A pathologic complete response, which is associated with favorable prognosis, generally requires at least 6 cycles of neoadjuvant chemotherapy in breast carcinoma.^3^ However, because endometrial carcinoma may progress if chemotherapy is prolonged, the endometrial carcinoma was treated first, followed by completion of the neoadjuvant chemotherapy for breast carcinoma and subsequent breast surgery. Consequently, postoperatively, beginning in June 2024, the patient received 3 more TCH cycles administered at 3-week intervals. Follow-up mammogram showed no breast mass and no axillary lymphadenopathy.

**Figure 5.**
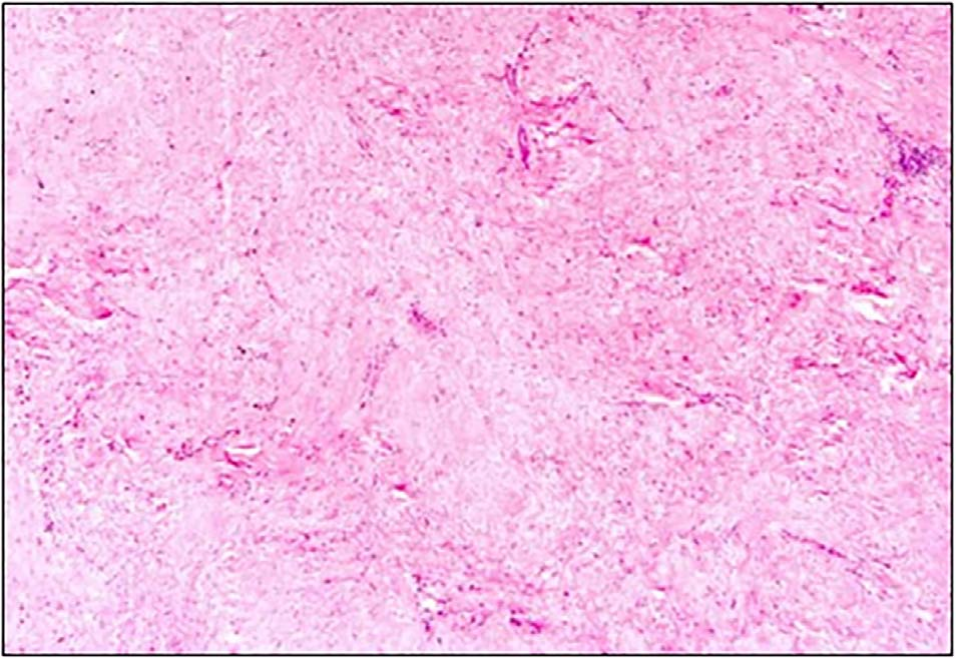
Hematoxylin and eosin stain of left breast tissue shows pathologic complete response after neoadjuvant chemotherapy.

In August 2024, the patient underwent left modified radical mastectomy with axillary dissection, achieving pathologic complete response (Figure 5). Adjuvant radiotherapy of 40 Gy in 15 fractions to the left axillary lymph nodes, left internal mammary lymph nodes, and left supraclavicular lymph nodes and 1 year of HER2-targeted therapy with trastuzumab 6 mg/kg for 12 cycles (each cycle every 2 weeks) were planned (Table 2). The patient is currently on regular follow-up and is doing well.

**Table 2.** Sequence of Clinical, Diagnostic, and Treatment Events

Time Point	Clinical/Diagnostic/Treatment Event	Diagnostic Findings	Outcome
January 2024	Patient presented with postmenopausal bleeding and abdominal pain MRI	19 × 15 × 11-mm polypoidal endometrial lesion	Suspicion of endometrial carcinoma
January 2024	PET/CT	FDG uptake in endometrial cavity (SUV 19.1), left breast nodules (largest 7 mm; SUV 1.8), and left axillary lymph nodes (largest 12 × 8 mm; SUV 2.1)	Suspicion of dual malignancies
January 2024	Mammography FNAC of axillary lymph nodes	Mammography: BI-RADS category 1 FNAC: positive for adenocarcinoma	Suspicion of metastatic breast carcinoma
January 2024	Immunohistochemistry analysis	Endometrial carcinoma: ER/PR positive, PAX8 positive Axillary nodes: HER2 positive, GATA3 positive	Confirmed dual primary malignancies
February to April 2024	Neoadjuvant chemotherapy, 3 cycles, TCH regimen Response assessment with MRI pelvis and sonomammogram	MRI pelvis: Reduced lesion size, partial myometrial infiltration Sonomammogram: No mass lesions in both breasts, no axillary lymphadenopathy	Endometrium: Good response Breast lesion: Complete response
May 2024	Radical hysterectomy, bilateral salpingo-oophorectomy, pelvic lymph node dissection	Endometrioid carcinoma, FIGO stage IA2	Curative resection
June to August 2024	Adjuvant chemotherapy, 3 cycles, TCH regimen Follow-up mammogram	Bilateral breast parenchyma appears normal, no axillary lymphadenopathy	Complete response
August 2024	Left modified radical mastectomy, axillary dissection		Pathologic complete response
September 2024	Radiotherapy of 40 Gy in 15 fractions to the left axillary lymph nodes, left internal mammary lymph nodes, and left supraclavicular lymph nodes		
2024 to 2025	Targeted therapy	HER2-directed therapy planned for 1 year	Long-term management ongoing

BI-RADS, Breast Imaging Reporting and Data System; ER, estrogen receptor; FDG, fluorodeoxyglucose; FIGO, International Federation of Gynaecology and Obstetrics; FNAC, fine needle aspiration cytology; GATA3, GATA binding protein 3; HER2, human epidermal growth factor receptor 2; MRI, magnetic resonance imaging; PAX8, paired box gene 8; PET/CT, positron emission tomography/computed tomography; PR, progesterone receptor; SUV, standard uptake value; TCH, docetaxel, carboplatin, trastuzumab.

## DISCUSSION

Cases reported by Yeh et al, Banimostafavi et al, and Watanabe and Yonekawa state that the coexistence of synchronous primary cancers in a single patient is infrequent and requires careful differentiation from metastatic disease.^4-6^ According to the Warren and Gates definition of synchronous primary cancers, both tumors must be malignant, each must be distinct, and the possibility of one being a metastasis of the other must be excluded.^7^ The Surveillance, Epidemiology, and End Results Program defines multiple primary cancers as synchronous if a second primary tumor occurs within 2 months of the initial diagnosis.^8^ Distinct histologic and immunohistochemical profiles were key in confirming synchronous primaries in our patient. Clear histologic differences between the tumors facilitated the diagnosis of 2 separate primary cancers.

Rhoades et al demonstrated that the risk of developing a second primary cancer was higher in patients with endometrial carcinoma vs the general population (standardized incidence ratio=1.05, 95% CI 1.03-1.07).^9^ Annegers and Malkasian demonstrated that the relative risk of developing breast cancer following endometrial carcinoma was 1.3 times that of the general population.^10^ However, this increased risk was limited to patients who shared established breast cancer risk factors, particularly nulliparity and, to a lesser extent, obesity. In contrast, parous and nonobese women with endometrial carcinoma did not demonstrate an elevated risk of developing subsequent breast cancer.^10^

Key and Pike demonstrated that disruption of the estrogen-progesterone balance increases endometrial cancer risk.^11^ Azziz reported that obesity contributes to breast and endometrial cancer through the peripheral aromatization of adrenal androgens into estrogens,^12^ while Ricceri et al noted that tamoxifen therapy is associated with a modest increase in the risk of endometrial carcinoma.^13^

Genetic alterations play a pivotal role. BRCA1 and BRCA2 mutations are linked to breast, ovarian, uterine, and pancreatic cancer, as well as melanoma. These mutations occur more frequently in patients with a family history of cancer than in patients with sporadic breast cancer.^14^ Lynch syndrome, caused by mutations in DNA mismatch repair genes (MLH1, MSH2, MSH6, PMS2, and EPCAM), predisposes to endometrial and colorectal cancers, with MLH1 and MSH2 mutations showing higher penetrance and earlier onset.^15^ Connelly et al noted that the PTEN, PALB2, TP53, BARD1 and ATM genes are associated with an increased risk of breast carcinoma.^16^ Accurate histopathologic and molecular profiling is therefore essential for personalized management.

PET/CT plays a critical role in staging both cancers. In a meta-analysis by Kakhki et al, 18F-FDG PET/CT showed moderate accuracy (sensitivity of 82% and specificity of 90%) for detecting primary endometrial carcinoma and high accuracy (sensitivity of 96% and specificity of 95%) for identifying distant metastases.^17^ In the meta-analysis by Iacovitti et al, the pooled prevalence of breast incidental uptake detected by 18F-FDG PET/CT was 0.5%, with a pooled malignancy risk of 60.9%.^18^ FDG PET/CT is not primarily used for breast screening, but incidentally detected breast uptake is likely to represent malignancy. Further, pooled data from Kasem et al showed that 18F-FDG PET/CT had low sensitivity (52.2%) but high specificity (91.6%) for detecting axillary lymph node metastases.^19^ Because of this limited sensitivity—particularly for micrometastatic disease—PET/CT should not be used as the sole modality for axillary nodal evaluation and cannot replace sentinel lymph node biopsy.^19^

Treatment strategies must prioritize the malignancy that dictates overall prognosis. Molecular and genetic profiling guides targeted therapies and reduces overtreatment. A multidisciplinary approach is indispensable to coordinate surgical, chemotherapeutic, and radiotherapeutic interventions while preserving patient quality of life.

## CONCLUSION

This case highlights the successful management of synchronous, early-stage endometrial endometrioid carcinoma and an occult HER2-positive breast carcinoma that presented uniquely with axillary nodal metastasis despite negative breast imaging. The case emphasizes the invaluable role of systemic FDG PET/CT and comprehensive immunohistochemical evaluation in detecting occult primary tumors. To mitigate the risk of endometrial cancer progression during neoadjuvant chemotherapy, a strategic *sandwich* approach was utilized. This tailored strategy was therapeutically efficacious for the patient.

## Notes

*This article meets the Accreditation Council for Graduate Medical Education and the American Board of Medical Specialties Maintenance of Certification competencies for Patient Care and Medical Knowledge.*
